# The evaluation of low cut‐off index values of Elecsys^®^ HIV combi PT assay in predicting false‐positive results

**DOI:** 10.1002/jcla.23503

**Published:** 2020-08-25

**Authors:** Zhuoyun Tang, Yu Gou, Keyi Zhang, Zhongyi Zhao, Yinhao Wei, Dongdong Li, Li Chen, Chuanmin Tao

**Affiliations:** ^1^ Department of Laboratory Medicine West China Hospital of Sichuan University Chengdu China; ^2^ West China Second University Hospital of Sichuan University Chengdu China; ^3^ Clinical Lab Wenjiang Zhongyi Hospital Chengdu China

**Keywords:** cut‐off index, Elecsys^®^ HIV combi PT assay, false‐positive, HIV

## Abstract

**Objective:**

To analyze the results of different cut‐off index (COI) values of Elecsys^®^ HIV combi PT assay and to assess the role of COI in reducing the frequency of false‐positive results.

**Methods:**

We conducted a retrospective study of samples analyzed by Elecsys^®^ HIV combi PT assay, a 4th‐generation ECLIA, between 2016 and 2017. A total amount of 379 122 samples were collected for HIV (Human Immunodeficiency Virus) screening.

**Results:**

A total of 379 122 samples were analyzed. 2528 (0.67%) were positive by Elecsys^®^ HIV combi PT. Of these, 468 were false‐positive results, and most of them (94.87%) were in samples with 1 < COI < 15. The false‐positive rate was 0.12%. Patients with false‐positive samples were more distributed in elder (*P* < .001) and female (*P* < .001) than true‐positive specimens. The median COI in true‐positive specimens was (385.20), which is significantly higher than false‐positive specimens (2.08). The consistency between Elecsys^®^ HIV combi PT assay and 3rd‐generation and positive predictive value (PPV) increased with higher COI values. Cancer, infection, and neurological diseases were considered the potential confounding factors of HIV false‐positive results (19.44%, 11.11%, and 6.62%, respectively).

**Conclusion:**

Samples with low COI values, especially those contain confounding factors, need to be further scrutinized to determine whether the confounding factors may cause false‐positive problem. In addition, the hypothesis that low COI values may predict false‐positive results is valid.

## Graphical abstract

A retrospective study carried out to analyze the results of different cut‐off index (COI) values of Elecsys^®^ HIV combi PT assay. We found that the samples with low COI values, especially those containing confounding factors, need to be further scrutinized to determine whether the confounding factors may cause a false‐positive problem. The hypothesis that low COI values may predict false‐positive results is valid.

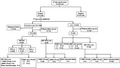

## 1 INTRODUCTION

HIV/AIDS continues to pose a serious burden of morbidity and mortality globally. As reported by WHO, till 2018, there were more than 37.9 million people living with HIV and only 75% of them were aware of their infection status.^1^ The spread of HIV/AIDS is alarming to many countries, including China, where the HIV prevalence is <0.1%.^2^ In recent years, China has strengthened advocating to improve public awareness of HIV/AIDS. The implementation has gone all out to achieve the UNAIDS/WHO 90‐90‐90 target, namely 90% of infected people should know their status, 90% of diagnosed cases should be on antiretroviral medication, and 90% of those being treated should have fully suppressed viral load by 2020.^3^

Novel 4th‐generation screening and confirmatory assays are now commercially available and have been incorporated into new diagnostic algorithms.^4^, ^5^ The 4th‐generation immunoassay can identify HIV‐1 p24 antigen, anti‐HIV IgM, and IgG antibodies simultaneously with sufficient sensitivity and specificity, thus narrowing the “window period” into approximately two weeks, and enabling the detection of acute and early HIV infection.^6^, ^7^ Due to the extremely high sensitivity of these techniques, the positive predictive value (PPV) in settings with a low prevalence of HIV may not be optimal, thus potentially leading to false‐positive results, arousing unnecessary concerns and interfering the progress of clinical diagnosis.^8^, ^9^ This study was carried out to analyze results for different COI values and the use of COI to distinguish between false‐positive and true‐positive results in a low‐prevalence setting through the presented hypothesis, we suggest that low COI values could predict false‐positive HIV results.

## 2 MATERIALS AND METHODS

### 2.1 Settings

We conducted a retrospective study in a large general teaching hospital with 4300 beds and a catchment population of approximately 16.33 million inhabitants in Sichuan, China. We included serum samples delivered to our laboratory, from inpatients and outpatients, for HIV testing between 2016 and 2017.

### 2.2 The screening tests

Samples were first analyzed using Elecsys^®^ HIV combi PT assay, performed on the MODULAR ANALYTICS E170 or Cobas e 601 platform (Roche Diagnostics), following the manufacturer's instructions. Samples were considered as reactive (COI ≥ 1.0), borderline (0.9 ≤ COI < 1.0), or non‐reactive (COI < 0.9). All initially reactive or borderline samples were redetermined in duplicate with 3rd‐generation assay—colloidal gold method—Anti‐HIV (Livzon Diagnostics Inc) or 3rd‐enzyme‐linked immunosorbent assay (ELISA) which was run on the TECAN (freedom evolyzer, Switzerland) according to the national guide of China. Samples were considered repeatedly reactive (control line and test line both appear/ COI ≥ 0.9 in either of the determinations) and non‐reactive (only control line appear or COI < 0.9) by 3rd‐generation assay. These assays were all conducted following the manufactures’ instructions.

### 2.3 The supplemental tests

Repeatedly, reactive samples must be confirmed according to recommended confirmatory algorithms. Western Blot HIV Blot 2.2 (MP Diagnostic) detects IgG antibodies specific to viral antigens. On the basis of manufacturer's criteria, the results of individual specimens were reported as positive (the presence of at least two bands, including two *env* bands, or one *env* band plus p24 band), indeterminate (reactivity to any of the bands but not compatible with the criteria for a positive interpretation), or negative (the absence of any of the specific bands). As a negative result only means that no IgG antibody detected, patients with indeterminate or negative results required further tests or follow‐up. HIV p24 antigen and HIV‐1 viral load could both detect early infection and narrow the gap of window period.^10^ HIV p24 antigen quantitative test was performed by Elecsys HIV Ag assay (Roche Diagnostics) on MODULAR ANALYTICS E170 or Cobas e 601 platform. Samples were considered as reactive (COI ≥ 1.0), borderline (0.9 ≤ COI < 1.0), or non‐reactive (COI < 0.9). Cobas AmpliPrep/Cobas TaqMan HIV‐1 test (Roche Diagnostics) was applied for HIV‐1 RNA detection, with lower detection limit of 15 CPs/mL. Samples were classified into three groups (>5000 CPs/mL, ≤5000 CPs/mL, and below detection limits).

Samples were considered as confirmed positive if they were reactive by immunoblot, HIV‐RNA, or follow‐up. HIV p24 Ag was used to help determine the infection status.

### 2.4 Blocking test

With sufficient sample size, a total amount of 193 samples with initially and repeatedly reactive, uncertain, or negative in antibody confirmation tests stepped into blocking test. HIV Ag/Ab was rerun, before and after blocking the samples with 40 μL Heterophilic Blocking Reagent (HBR, 20 μg/mL) added to each sample and reacted for 1 hour under room temperature.

### 2.5 Statistical analysis

SPSS 23.0 software was used for statistical analysis, and OriginPro 2016 was used for plotting. Quantitative data were expressed as median and interquartile range (IQR). Comparisons between continuous variables were made using the *t* test or variance analysis, depending on the normality of the distribution. If not, the non‐parametric test was adopted for analysis. The data were counted by *chi‐square* test or *Fisher's* exact probability test. A *P*‐value less than or equal to .05 was considered significant.

## 3 RESULTS

### 3.1 The distribution of true‐positive and false‐positive specimens

**FIGURE 1:**
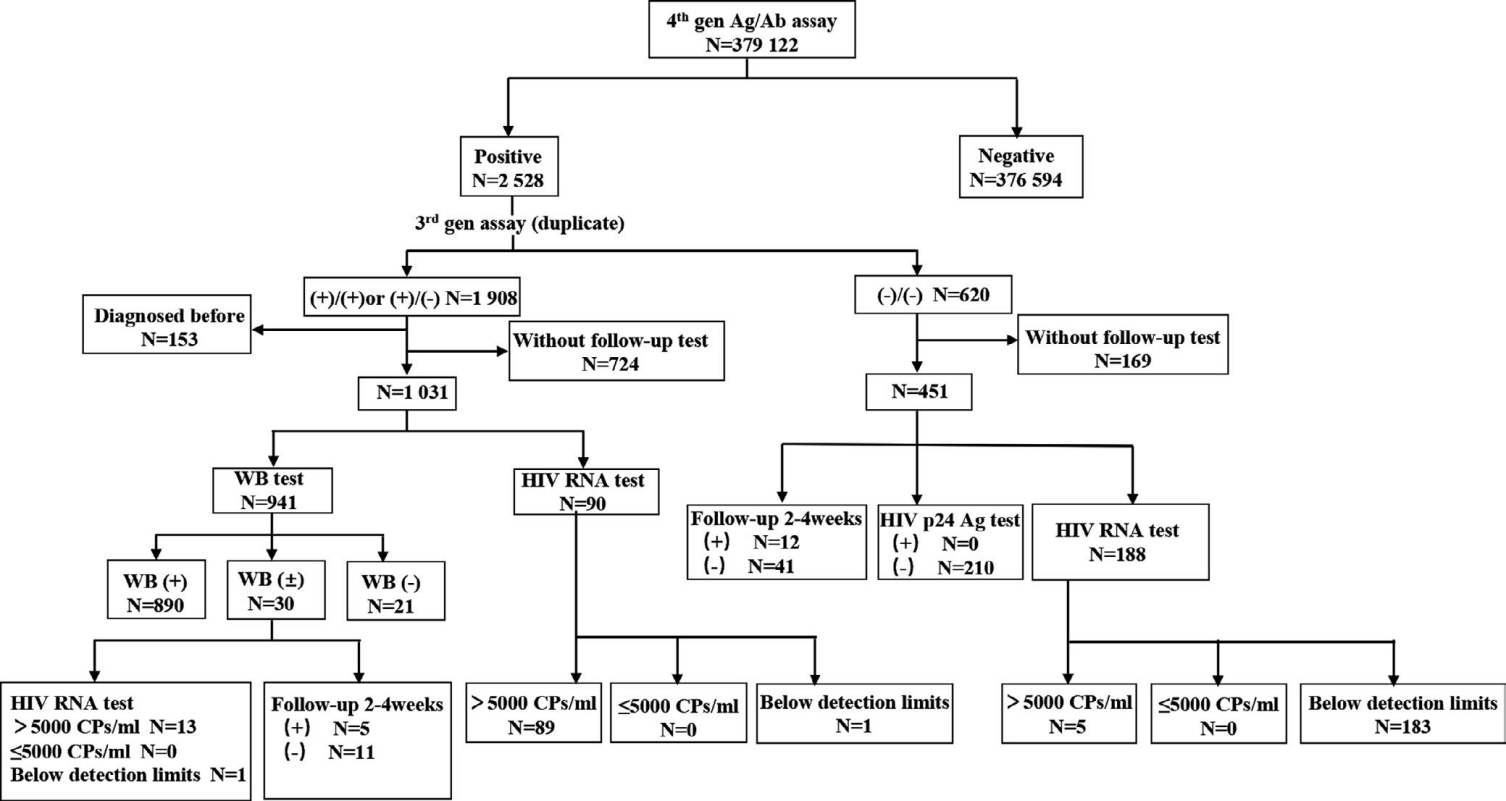
The distributions of the results of the Elecsys® HIV combi PT assay

A total of 379 122 samples were analyzed by Elecsys^®^ HIV combi PT according to the recommended algorithm by Chinese Centers for Disease Control and Prevention (CDC), and the distribution of samples by results is shown in Figure 1. Briefly, 2528 samples (0.67%) were initially reactive by Elecsys^®^, and 1908 (75.47%) of the 2528 samples were confirmed reactive. After ruling out 893 samples that have been lost in follow‐up, samples selected for WB/RNA/follow‐up according to the algorithm. At last, 1167 samples with true‐positive results (including 153 diagnosed before) and 468 samples with false‐positive results were reported from 2528 initially reactive samples. It is worth noting that the false‐positive rate accounted for 0.12%.

Compared features of true‐positive group, false‐positive group was prone to be older [52 (37‐65) vs 45 (31‐57), *P* < .001] [median (IQR)]. Notably, the proportion of male in false‐positive group was lower than that in true‐positive group [54.06% (253/468) vs 81.36% (825/1014), *P* < .001]. There was no significant difference in terms of ethnicity, educational level, marital status, and occupation.

### 3.2 COI analysis

**FIGURE 2:**
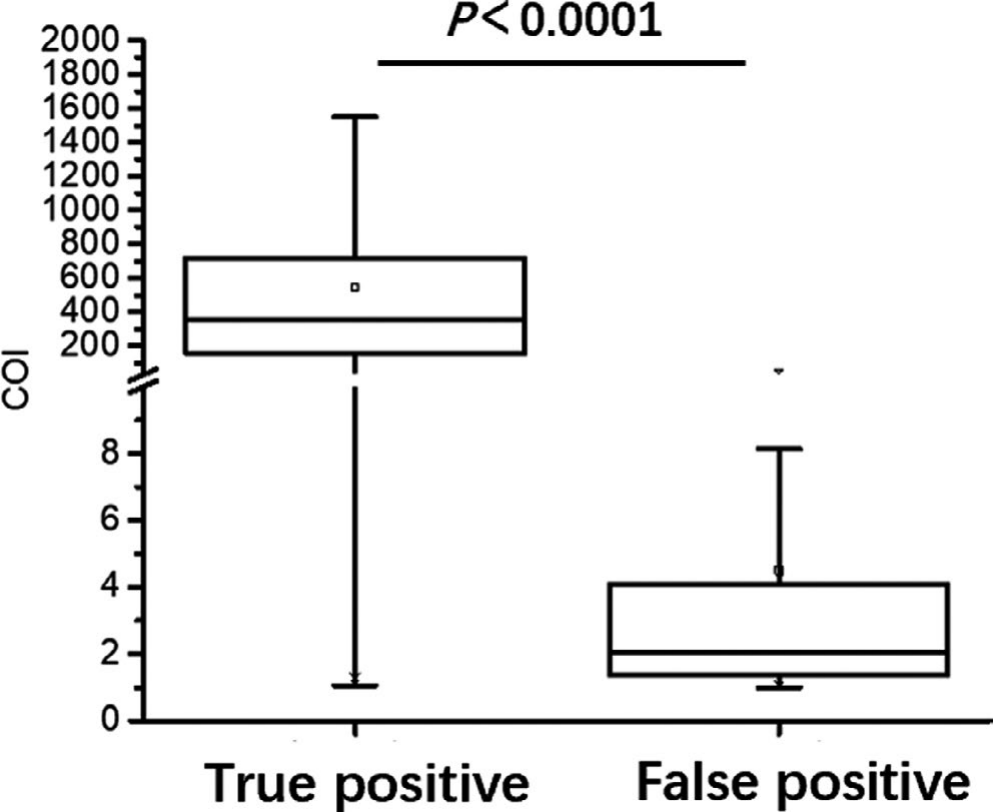
The COI values of the true‐positive specimens and the false‐positive specimens

The median COI value in true‐positive group was 385.20 (IQR:197.60‐769.60), while value in false‐positive group was 2.08 (IQR:1.36‐4.17), with statistically significant difference (*P* < .0001) (Figure 2).

Nearly, all (94.87%) of the false‐positive results had a COI between 1 and 15, and 434 out of 468 false‐positive specimens were non‐reactive by 3rd‐generation assay. COI ranged from 15 to 50 in 22 false‐positive samples, and only two samples fluctuated at 50‐100 COI, with value of 57.84 as the peak in all false‐positive samples. The consistency and PPV showed different trends with varied COI values. When COI value climbed, the consistency and PPV increased simultaneously, while the false‐positive specimens decreased (Table 1).

**TABLE 1:** Consistency and PPV of different COI values

Screening tests (Elecsys^®^ HIV Combi PT assay)		Retesting by 3rd‐generation			PPV (%)
COI Values	No.	Reactive (FP)	Non‐reactive (FP)	Consistency (%)	
1~	425	5 (4)	420 (420)	1.18	0.24
15~	46	23 (14)	23 (12)	50.00	43.48
50~	91	86 (16)	5 (2)	94.51	80.2
100~	357	356 (0)	1 (0)	99.72	100
300~	432	431 (0)	1 (0)	99.77	100
800~	284	283 (0)	1 (0)	99.65	100
Total	1635	1184 (34)	451 (434)	72.42	71.38

Abbreviation: FP, false‐positive.

The sensitivity and specificity for Elecsys^®^ HIV Combi PT assay were 100% (95% CI: 100‐100) and 99.88% (95% CI: 99.78‐99.98), respectively. ROC analysis showed the COI valued at 52.74 with 0.998 AUC (*P* < .0001). Correspondingly, the false‐positive rate declined to 0.01% and PPV rose to 99.92% (95% CI: 99.75‐100).

### 3.3 The clinical characteristics of false‐positive samples and blocking test

The three leading clinic diagnoses among false‐positive patients were cancer, infection, and neurological diseases, with the proportions of 19.44%, 11.11%, and 6.62%, respectively (Table 2). The 91 cancer samples consisted of adenocarcinoma, invasive cancer, tumor metastasis, and advanced tumor.

**TABLE 2:** Clinical characters of false‐positive specimens

Clinic diagnosis	No.	Proportion（%）
Cancer	91	19.44
Infection	52	11.11
Neurological diseases	31	6.62
Cardiovascular diseases	29	6.20
Just examination	26	5.56
physical examination	22	4.70
Orthopedic diseases	21	4.49
Ophthalmic diseases	20	4.27
Mental diseases	17	3.63
Preoperative examination	13	2.78
Lithiasis	12	2.56
Autoimmune diseases	11	2.35
Liver cirrhosis	11	2.35
Chronic obstructive pulmonary disease	10	2.14
Disease of digestive tract	9	1.92
Renal insufficiency	7	1.50
Others	86	18.38
Total	468	100

Ruling out 60 of 193 samples showed negative results already by retesting HIV Ag/Ab, so we blocked the remaining 133 samples then retested HIV Ag/Ab. The results indicated 16 out of 133 samples turned to be negative after blocking. The median COI of 16 samples changed from 1.46 (IQR:1.67‐5.97) to 0.82 (IQR:0.39‐0.89) with no significant difference (*P* > .05). Moreover, the clinical diagnosis of the 16 turning negative patients has no characteristic.

## 4 DISCUSSION

The 4th‐generation assay is capable for detecting both HIV‐1 p24 antigen and antibody simultaneously, which can narrow the “window period” into about two weeks, making it widely used as screening assay.^11^ However, Western blot used as supplemental assay can only detect the HIV‐1 antibody. West China Hospital uses the Elecsys^®^ HIV combi PT assay for screening, and WB, HIV‐RNA, and follow‐up visit as confirmation. The HIV infection rate of the included participants in this study was 0.31% (1167/379 122), which indicated a relatively low HIV prevalence in Chengdu region.

The Elecsys^®^ HIV combi PT assay, a 4th‐generation assay, is currently recognized and recommended as HIV screening test internationally. It has a special pre‐treatment of samples to release antigen and can evaluate the diagnostic performance.^12^, ^13^ According to our study, the specificity and sensitivity of the Elecsys^®^ HIV combi PT were 99.88% and 100%. The false‐positive rate and positive predictive value were 0.12% and 71.38%. The results were similar to previous studies that the Elecsys^®^ HIV combi PT performed well in specificity (>99.50%) and sensitivity (nearly 100%) (Table 3). Considering all these merits of the 4th‐generation assay, it is more appropriate for routine clinical screening and HIV early infection screening in high‐risk groups.^17^

**TABLE 3:** Diagnose performance of Elecsys^®^ HIV Combi PT assay of this study and different studies before

Authors	Sensitivity（%）	Specificity（%）	FP（%）	PPV（%）
This study	100 (100‐100)	99.88 (99.78‐99.98)	0.12	71.38 (70.68‐75.00)
Annette Blaich^8^	NP	99.70（99.60‐99.90）	NP	71.80（57.70‐85.90）
Denise L.Uettwiller‐Geiger^14^	100（99.75‐100）	99.94（99.85‐99.98）	NP	NP
Eun Young Song^15^	100（98.20‐100）	99.50（99.00‐99.90）	0.49	NP
Yongming Liu^16^	100 (100‐100)	99.93 (99.91‐99.95)	0.07	82.21 (78.54‐85.88)

Abbreviations: FP, false‐positive; NP, not provided; PPV, positive predictive value.

In this study, false‐positive specimens showed some similarities. The COI value was significantly lower in the false‐positive group than that in the true‐positive group. Nearly, all false‐positive samples were distributed at a low level of COI (1‐15), and a higher COI value accompanied by fewer false‐positive samples. Results also showed that most patients with false‐positive results were elders and females, which was consistent with previous studies.^17^, ^18^ Recent studies revealed that many factors, such as cancers, rheumatoid factors, autoimmune diseases, and pregnancy, might lead to poor specificity in HIV screening by the 4th‐generation assay and cause an indeterminate result for the WB test.^19^, ^20^, ^21^, ^22^, ^23^, ^24^ More elderly patients in the false‐positive group are more likely to suffer from underlying diseases. These diseases and some potential confounding substances in patients may blamed for some false‐positive results. Although follow‐up visit and use of HBR for blocking can eliminate interference to certain extent, proceeding HIV‐RNA test as soon as possible, the history of epidemiology and confounding factors should also be taken into consideration. Besides, it is of vital importance to enhance the communication between the laboratory and clinic.

Our study is subject to limitations. First, it is a retrospective study and some data were missing. But this will be perfected by our follow‐up study. Second, the data came from one hospital and it cannot be generalizable to the entire local population. Therefore, multi‐center clinical experiments should be conducted to further confirm the results.

In conclusion, samples with low COI values, especially those with confounding factors, need to be further censored to determine whether confounding factors could have an effect on the results or not. In addition, the hypothesis that low COI values may predict false‐positive results is valid.

## 5 ETHICAL CONSIDERATIONS

The study was conducted in full compliance with the principles of the Helsinki Declaration and local regulations. The study protocol was approved by ethics committee of the West China Hospital of Sichuan University. Exemption for obtaining informed consents from subjects was granted as a retrospective analysis of routinely collected programmatic data, and there was no direct contact with patients.

## References

[1] Organization WHO, HIV/AIDS . http://www.who.int/news‐room/fact‐sheets/detail/hiv‐aids. Accessed August 16, 2019.

[2] NCAIDS, NCSTD, China CDC . Update on the AIDS/STD epidemic in China in April, 2018.

[3] UNAIDS . 90–90‐90 an ambitious treatment target to help end the AIDS epidemic Geneva: UNAIDS. http://www.unaids.org/sites/default/files/media_asset/90‐90‐90_en_0.pdf. Accessed November 25, 2019.

[4] Liu P , Jackson P , Shaw N , et al. Spectrum of false positivity for the fourth generation human immunodeficiency virus diagnostic tests. AIDS Res Ther. 2016;13:1. 26734067 10.1186/s12981-015-0086-3 PMC4700595

[5] Chacón L , Mateos ML , Holguín Á . Relevance of cutoff on a 4th generation ELISA performance in the false positive rate during HIV diagnostic in a low HIV prevalence setting. J Clin Virol. 2017;92:11‐13. 28501753 10.1016/j.jcv.2017.04.014

[6] Alexander TS . Human immunodeficiency virus diagnostic testing: 30 years of evolution. Clin Vaccine Immunol. 2016;23(4):249‐253. 26936099 10.1128/CVI.00053-16 PMC4820517

[7] Stone M , Bainbridge J , Sanchez AM , et al. Comparison of detection limits of 4th generation combination HIV antigen/antibody, p24 antigen and viral load assays on diverse HIV isolates. J Clin Microbiol. 2018;56:e02045‐e2117. 29793968 10.1128/JCM.02045-17 PMC6062817

[8] Blaich A , Buser A , Stöckle M , et al. Specificity of two HIV screening tests detecting simultaneously HIV‐1 p24 antigen and antibodies to HIV‐1 and ‐2. J Virol Methods. 2017;11(249):143‐146. 10.1016/j.jviromet.2017.09.005 28893550

[9] Shima‐Sano T , Yamada R , Sekita K , et al. A human immunodeficiency virus screening algorithm to address the high rate of false‐positive results in pregnant women in Japan. PLoS One. 2010;5(2):e9382. 20186348 10.1371/journal.pone.0009382 PMC2826425

[10] Wang T , Li D , Yan K , et al. Performance evaluation of a new fourth‐generation HIV Ag/Ab combination electrochemiluminescence immunoassay ‐ evaluation of a new HIV assay. Int J STD AIDS. 2014;25(4):267‐272. 23970655 10.1177/0956462413499909

[11] Mahajan VS , Pace CA , Jarolim P . Interpretation of HIV serologic testing results. Clin Chem. 2010;56(10):1523‐1526. 20876778 10.1373/clinchem.2009.139535

[12] Tao CM , Cho Y , Ng KP , et al. Validation of the Elecsys^®^ HIV combi PT assay for screening and reliable early detection of HIV‐1 infection in Asia. J Med Virol. 2013;58(1):221‐226. 10.1016/j.jcv.2013.05.012 23809476

[13] Mühlbacher A , Schennach H , Helden JV , et al. Performance evaluation of a new fourth‐generation HIV combination antigen–antibody assay. Med Microbiol Immunol. 2013;202(1):77‐86. 22706797 10.1007/s00430-012-0250-5 PMC3562432

[14] Uettwiller‐Geiger DL , Lessig M , An J , et al. Analytical and clinical performance evaluation of the Elecsys HIV combi PT Assay on the cobas e 602 analyzer for the diagnosis of human immunodeficiency virus. Am J Clin Pathol. 2019;151(4):377‐385. 30423023 10.1093/ajcp/aqy153 PMC6396746

[15] Song EY , Hur M , Roh EY , et al. Performances of four fourth‐generation human immunodeficiency virus‐1 screening assays. J Med Virol. 2012;84(12):1884‐1888. 23080491 10.1002/jmv.23423

[16] Liu Y , Li D , Wang T , et al. Clinical application evaluation of two fourth‐generation human immunodeficiency virus (HIV) screening assays in West China Hospital. J Clin Lab Anal. 2015;29(2):146‐152. 24797498 10.1002/jcla.21743 PMC6807122

[17] Rutstein SE , Ananworanich J , Fidler S , et al. Clinical and public health implications of acute and early HIV detection and treatment: a scoping review. J Int AIDS Soc. 2017;20(1):21579. 28691435 10.7448/IAS.20.1.21579 PMC5515019

[18] Wang L , Xiao Y , Tian XD , et al. HIV infection in Xi’an, China: epidemic characterization, risk factors to false positives and potential utility of the sample‐to‐cutoff index to identify true positives using Architect HIV Ag/Ab combo. Antimicrob Resist Infect Control. 2019;8:9. 30651975 10.1186/s13756-019-0463-0 PMC6329139

[19] Parker J , Carrasco AF , Chen J . BioRad BioPlex® HIV Ag‐Ab assay: incidence of false positivity in a low‐prevalence population and its effects on the current HIV testing algorithm. J Clin Virol. 2019;116:1‐3. 30981082 10.1016/j.jcv.2019.04.002 PMC6557666

[20] Vega LE , Espinoza LR . HIV infection and its effects on the development of autoimmune disorders. Pharmacol Res. 2018;129:1‐9. 29331332 10.1016/j.phrs.2018.01.005

[21] Jian L , Liang W , Zhang Y , et al. Systemic lupus erythematosus patient with false positive results of antibody to HIV: A case report and a comprehensive literature review. Technol Health Care. 2015;23(s1):S99‐S109. 26410337 10.3233/thc-150938

[22] Lavoie S , Caswell D , Gill MJ , et al. Heterophilic interference in specimens yielding false‐reactive results on the Abbott 4th generation ARCHITECT HIV Ag/Ab Combo assay. J Clin Virol. 2018;104:23‐28. 29704735 10.1016/j.jcv.2018.03.014

[23] Leslie S , Koert R , Erwan P , et al. Accounting for false positive HIV Tests: is visceral leishmaniasis responsible? PLoS One. 2015;10(7):e0132422. 26161864 10.1371/journal.pone.0132422 PMC4498794

[24] Adhikari EH , Devin M , Donna G , et al. Diagnostic accuracy of fourth‐generation ARCHITECT HIV Ag/Ab Combo assay and utility of signal‐to‐cutoff ratio to predict false‐positive HIV tests in pregnancy. Am J Obstet Gynecol. 2018;219(4):408.e1‐408.e9. 29913173 10.1016/j.ajog.2018.06.008

